# *Sarcocystis moreliae* sp. nov. in the imported green tree python *Morelia* cf. *viridis* (Reptilia, Pythonidae)

**DOI:** 10.3389/fvets.2023.1211522

**Published:** 2023-07-04

**Authors:** Ondřej Máca, David González-Solís

**Affiliations:** ^1^Department of Pathology and Parasitology, State Veterinary Institute Prague, Prague, Czechia; ^2^Department of Zoology and Fisheries, Faculty of Agrobiology, Food and Natural Resources, Czech University of Life Sciences Prague, Prague, Czechia; ^3^El Colegio de la Frontera Sur, Chetumal, Mexico

**Keywords:** Indonesia, *Sarcocystis*, reptile, intermediate host, molecular characterization, new species

## Abstract

Species of *Sarcocystis* use various vertebrates as intermediate or definitive hosts in their life cycles. One of these is snakes, whose role as intermediate hosts for these protozoans is scarce; in fact, there are six records, but only three with molecular characterization. An imported green tree python was involved in the morphological and molecular characterization (four loci) of a new species of *Sarcocystis* localized in skeletal muscles. *Sarcocystis moreliae* sp. nov. has a type 1 sarcocyst with a smooth wall and is genetically similar (at the *18S* rRNA gene) to two unnamed species of *Sarcocystis* found in *Lytorhynchus diadema* from Oman and *Varanus salvator macromaculatus* from Malaysia, but their detailed comparison is impossible. The new species showed lower similarity to its congeners in other loci (*28S* rRNA, ITS1, and *cox1*). This is the first morphological and genetic characterization of a *Sarcocystis* species in snakes of the genus *Morelia*, particularly *M. viridis*, using four loci, but more data are needed to fill the knowledge gap about snakes as intermediate hosts of *Sarcocystis*.

## Introduction

Among the protozoans of the genus *Sarcocystis* Lankester, 1882 are parasites of reptiles (avian and non-avian), mammals, and fish that act as intermediate (IHs) and/or definitive hosts (DHs) in an obligatory heteroxenous life cycle. To date, mammals harbor the vast majority of known species of *Sarcocystis* by acting as IHs (148) and DHs (56), followed by reptiles [IH: 23, DH: 26; see (1–3)]. However, most of those *Sarcocystis* species in reptiles have only been found in lizards and have been solely evaluated morphologically and morphometrically; they lack a molecular analysis and cannot be compared with other congeners.

In particular, two named and four unnamed species of *Sarcocystis* that use snakes as IHs have so far been reported worldwide [i.e., *S*. *atractaspidis* Parenzan, 1947 in *Atractaspis leucomelas* Boulenger, *S*. *pythonis* Tiegs, 1931 in *Morelia spilota* (Lacépède) (syns. *M. argus*, *Python spilotes*), and *Sarcocystis* spp. in *Crotalus durissus terrificus* Laurenti, *Lytorhynchus diadema* (Duméril, Bibron & Duméril), *Malpolon monspessulanus* (Hermann), and *Psammophis schokari* Forskall]. Of them, *S*. *atractaspidis* and *S*. *pythonis* are listed as *species inquirendae* by Dubey et al. (2) due to the absence of differential morphological descriptions and molecular characterization, while the sequence of *Sarcocystis* sp. in *C*. *d*. *terrificus* is absent from GenBank, and the three others were genetically analyzed and are compared herein with the new species.

The occurrence of *Sarcocystis* in snakes of the genus *Morelia* has been scarcely studied. In addition to the report of *S*. *pythonis* in *M. spilota* (Pythonidae) as an IH, there are also some records of DHs, such as *Sarcocystis* sp. in *M. spilota variegata* Gray from Australia (4), and *Sarcocystis* spp. (KC201639, KC201640) in *M. viridis* (Schlegel) from Germany (5).

The green tree python, *M. viridis*, is an arboreal snake inhabiting lowland and lower montane environments (0–2,000 m above sea level) from New Guinea Island up to north-eastern Australia, except the Bismarck Archipelago (6, 7). This python species feeds on lizards, mammals, and birds (8) and has become one of the most traded reptiles in the world (9). Since the molecular characterization of *Sarcocystis* species infecting and using snakes as IHs is scarce, and the role of *M*. cf. *viridis* in the life cycle of those protozoans is barely known, we report here the findings of sarcocysts in the skeletal muscles of a green tree python imported from Indonesia to the Czech Republic.

## Methods

In 2021, a green tree python (*Morelia* cf. *viridis*) was imported to the Czech Republic from an unknown locality in Southwestern Papua, Indonesia. Due to the unknown collection location and the various species of *Morelia* inhabiting this geographic area (see (10)), we decided to name it *M*. cf. *viridis*. In October 2022, this 2-year-old snake died following long-term health problems and poor body condition (0.3 kg in weight). The carcass was sent to the State Veterinary Institute (SVI) Prague for necropsy, where bacteriological and parasitological analyses were applied through blood smears and the examination of the intestine, stomach, subcutaneous tissue, body cavity, liver, and muscles from the heart, head, trunk, and tail. These examinations revealed mixed bacterial and parasitic infections (unpublished data), but only data on *Sarcocystis* are shown herein. Muscles were examined by wet mounts for the presence of sarcocysts under 10 − 100x objective magnifications of a Leica DM2500 LED optical microscope with a Leica DMC5400 digital camera and Leica Application Suite X microscope software (both Leica Microsystems, Wetzlar, Germany). For histology, muscles with sarcocysts were fixed in 10% formalin solution and tissue sections were stained with hematoxylin and eosin. All measurements are given in micrometers unless otherwise mentioned.

Individual sarcocysts (four isolates) or muscles with numerous sarcocysts (two isolates) were transferred to Eppendorf tubes for DNA extraction. Genomic DNA was extracted by glass bead disruption from six isolates collected from skeletal muscles (head, trunk, and tail) using the NucleoSpin tissue XS kit (Macherey-Nagel, Düren, Germany) according to the manufacturer’s recommendations. DNA was stored at –20°C until use in PCR assays of *18S* rRNA, *28S* rRNA, ITS1, and *cox1* loci. PCR and nested-PCR were carried out using primers for *18S* rRNA (11), *28S* rRNA (KL_P1F/KL_P2R) (12), ITS1 region (12), and *cox1* (SF1/SR5) (13). The amplification cycles for all genes were as follows: 95°C for 3 min, 5 cycles of 94°C for 45 s, 64°C for 60 s, 72°C for 90 s; followed by 30 cycles of 95°C for 30 s, 52–60°C for 30 s, 72°C for 1 min; and 72°C for 10 min. RNase/Dnase-free water was used as a negative control in each PCR test. PCR products were later analyzed by electrophoresis on a 1% agarose gel, visualized by ethidium bromide staining, and purified using the ExoSAP-IT™ Express PCR Product Cleanup Reagent Kit (Thermo Fisher Scientific) according to the manufacturer’s protocol. Cleaned amplicons were sequenced through the commercial company Eurofins Genomics (Ebersberg, Germany) using both forward and reverse primers. Nucleotide sequences of the four loci derived in this study have been deposited in GenBank^1^ under accession numbers (OQ296416–OQ296418, OQ295898). Sequences were analyzed and edited using FinchTV software (Geospiza Inc., Seattle, WA, United States) and compared with those of the valid species of *Sarcocystis* in the GenBank NCBI database using Basic Local Alignment Search Tool (BLAST; http://blast.ncbi.nlm.nih.gov/Blast.cgi). Alignment of sequences was performed using MAFFT software version 7.^2^

Phylogenetic relationships of *S*. *moreliae* sp. nov. at *18S* rRNA, *28S* rRNA, and ITS1 loci were analyzed using the MEGA11 software (14). Phylogenetic trees were inferred using the maximum-likelihood (ML) method, with the Hasegawa-Kishino-Yano parameter model with a gamma distribution rate and a proportion of invariant sites (HKY + G + I) according to the best-fit substitution model using Bayesian Information Criterion scores (BIC) in MEGA 11. Phylogenetic analysis involved 52 nucleotide sequences, with a total of 1,776 aligned nucleotide positions for *18S* rRNA, 28 sequences with 1,611 positions for *28S* rRNA, and 22 sequences with 1,714 positions for ITS1 loci. In the case of the *cox1* locus, phylogenetic methods used the ML method and a General Time Reversible model with a gamma distribution rate (GTR + G) because this best fitted the data involving 25 sequences in 1,038 positions. Bootstrap analyses were carried out using 1,000 replicates. Phylogenetic trees were constructed with sequences obtained in the present study along with reference sequences deposited in GenBank and rooted using the *Toxoplasma gondii* Nicolle et Manceaux, 1908 sequence and *S*. *neurona* Dubey, Davis, Speer, Bowman, de Lahunta, Granstrom, Topper, Hamir, Cummings et Suter, 1991 (ITS1).

## Results

**Figure 1:**
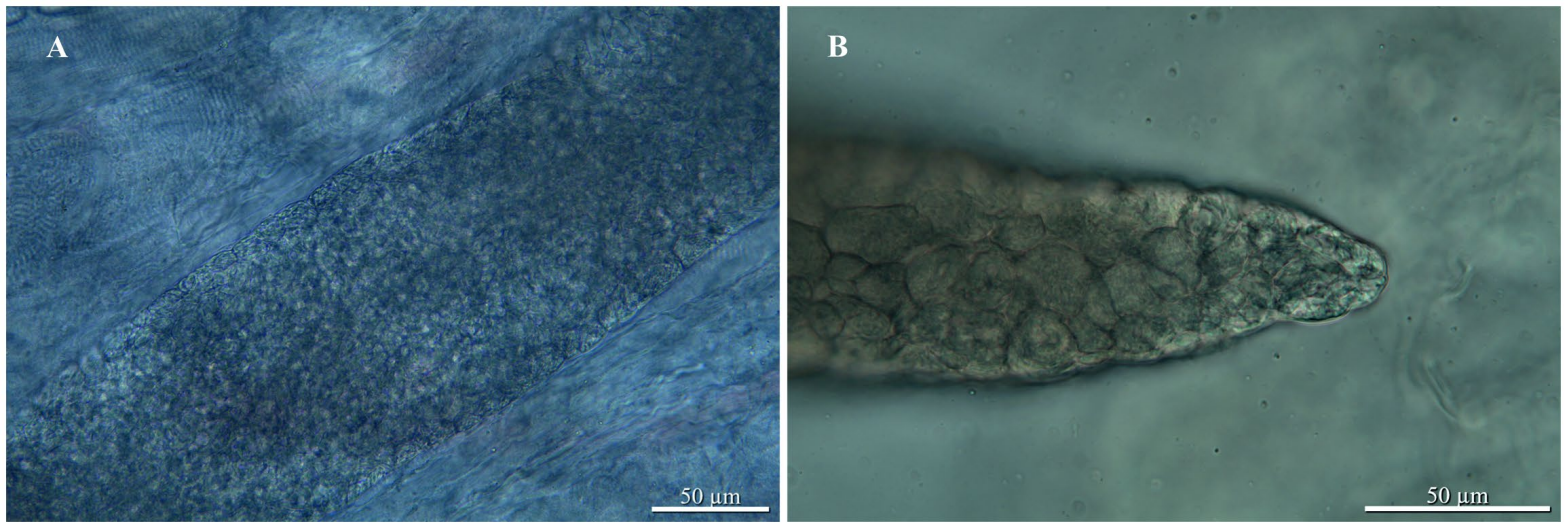
*Sarcocystis moreliae* sp. nov. from *Morelia* cf. *viridis*, light micrographs: **(A)** Sarcocyst in skeletal muscle of host; **(B)** Sarcocyst with septa.

**Figure 2:**
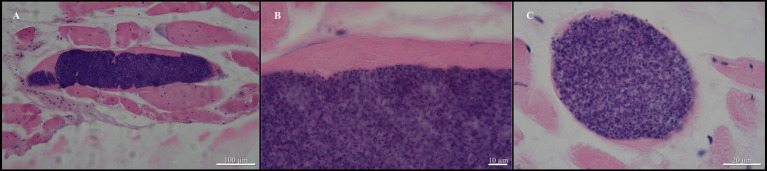
*Sarcocystis moreliae* sp. nov. from *Morelia* cf. *viridis*, hematoxylin, and eosin-stained histological sections of skeletal muscle. **(A)** Sarcocyst in skeletal muscle of host; **(B)** Same higher magnification showing sarcocyst wall without villar protrusions; **(C)** Transversal cut of sarcocyst showing bradyzoites.

Examination of the sole green tree python revealed the presence of sarcocysts in the skeletal muscles (head, trunk, tail; Figures 1A, 2A); muscles from the heart were negative for the presence of parasites. All sarcocysts were microscopic and divided into compartments by septa (Figure 1B). Based only on LM, the cyst wall appeared to be smooth without visible protrusions on its surface (Figures 2A−C), thus belonging to type 1, although determining the subtype is impossible without transmission electron microscopy studies. The largest sarcocyst measured 1,118 × 123 μm, with a wall thickness of 0.55 μm. The banana-shaped bradyzoites (*n* = 20) were 6.63–7.99 × 1.43–1.87 μm in size (Figures 2B,C). Histological examination showed that no visible inflammatory reaction occurred (Figures 2A−C).

### Taxonomic summary

Family Sarcocystidae Poche, 1913

*Sarcocystis moreliae* sp. nov. (Figures 1, 2).

Intermediate host: Green tree python *Morelia* cf. *viridis* (Serpentes, Pythonidae).

Definitive host: Unknown.

Original distribution: Southwestern Papua, Indonesia.

Deposited material: Symbiotype (frozen muscle with sarcocysts), and genomic DNA in an Eppendorf tube were stored at SVI Prague. GenBank sequences OQ296418 (*18S* rRNA gene), OQ296417 (*28S* rRNA gene), OQ296416 (ITS1 region), and OQ295898 (*cox1* gene).

ZooBank registration: To comply with the regulations set out in article 8.5 of the amended 2012 version of the International Code of Zoological Nomenclature (15), details of the new species have been submitted to ZooBank. The Life Science Identifier (LSID) for *Sarcocystis moreliae* sp. nov. is lsid:zoobank.org:pub:6A62F67A-CA7F-4938-AFFA-8EF0E62708F8.

Etymology: The specific epithet is derived from the generic name of its intermediate host, i.e., *Morelia*.

**Figure 3:**
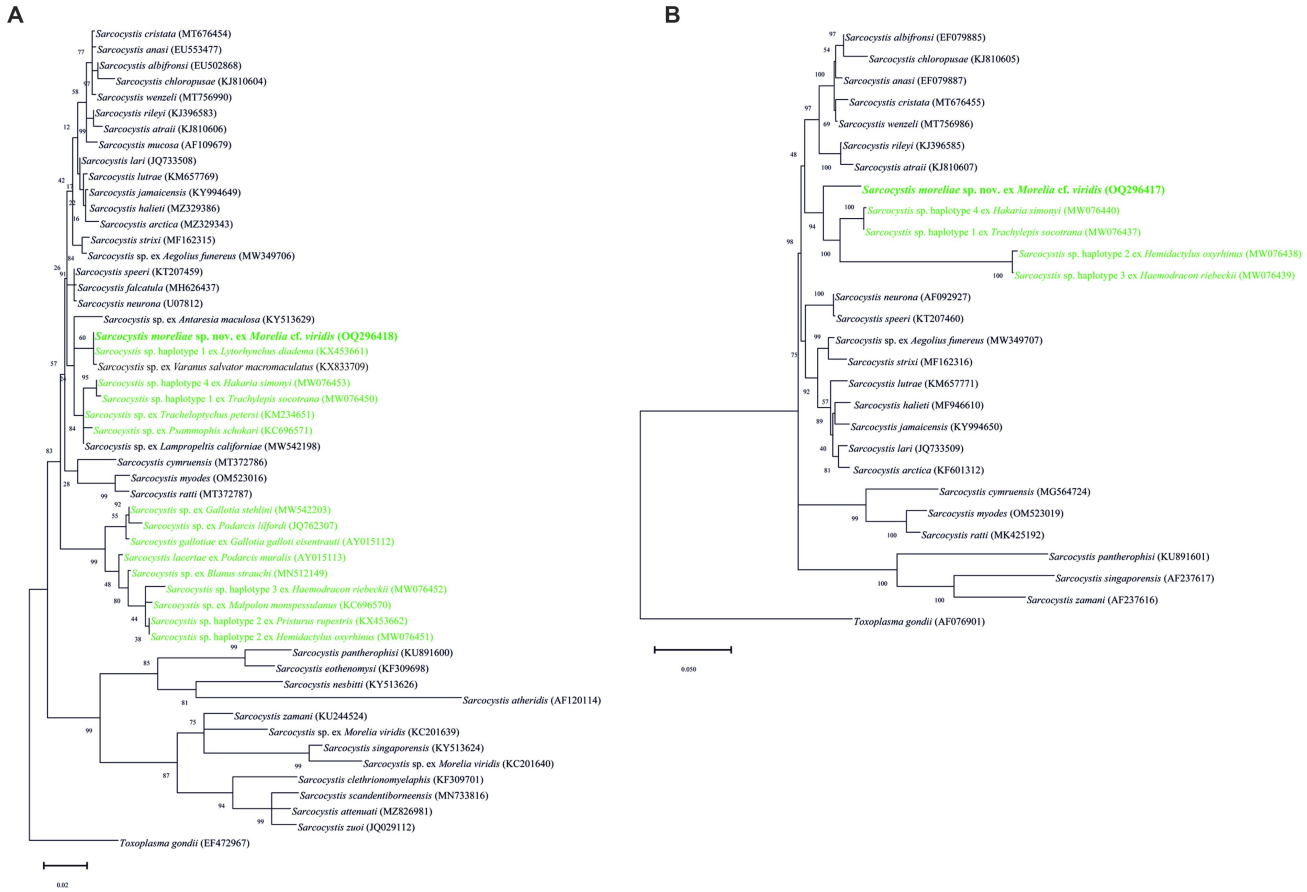
Phylogenetic trees of the species of *Sarcocystis*; those in green are for reptiles being used as intermediate hosts, based on **(A)** *18S* rRNA and **(B)** *28S* rRNA loci sequences. The numbers on phylogenetic trees represent bootstrap values based on 1,000 replications, while those after *Sarcocystis* species are GenBank accession numbers.

Molecular sequences of the *18S* rRNA, *28S* rRNA, ITS1, and *cox1* loci from six sarcocyst isolates were successfully obtained with no intraspecific variability. The *18S* rRNA representative sequence of *S*. *moreliae* sp. nov. showed query cover 31–100% with high similarity to two unnamed *Sarcocystis* species in the crowned leaf nose snake and in the common water monitor, respectively (see Table 1 for details). *Sarcocystis moreliae* sp. nov. formed a single clade with these two unnamed species (Figure 3A). The new species was also shown to be similar to *Sarcocystis* sp. in the California kingsnake, *S*. *speeri* Dubey and Lindsey, 1999 in the white-eared opossum, *S*. *lari* Prakas, Kutkienė, Butkauskas, Sruoga et Žalakevičius, 2014 in the white-tailed sea eagle, *S*. *halieti* Gjerde, Vikøren et Hamnes, 2018 in the red kite, *S*. *neurona* in the horse, and *S. lutrae* Gjerde et Josefsen, 2014 in the European otter (see Table 1 for details).

**Table 1:** Degree of similarity among *Sarcocystis moreliae* sp. nov with other *Sarcocystis* species retrieved from GenBank, by using for loci (*18S* rRNA, *28S* rRNA, ITS1, and *cox1*).

*Sarcocystis moreliae* sp. nov. (OQ296418; 1,661 bp)			
*18S* rRNA			
Species (sequence number; base pairs)	Similarity (query cover)^*^ %	Host species	Country
*Sarcocystis* sp. (KX453661; 588 bp)	100 (35)	*Lytorhynchus diadema*	Oman
*Sarcocystis* sp. (KX833709; 1,055 bp)	100 (63)	*Varanus salvator macromaculatus*	Malaysia
*Sarcocystis* sp. (MW542198; 532 bp)	99.25 (31)	*Lampropeltis californiae*	Spain
*S*. *speeri* (KT207459; 1,751 bp)	98.92	*Didelphis albiventris*	Argentina
*S*. *lari* (MF946588; 1,804 bp)	98.80	*Haliaeetus albicilla*	Norway
*S*. *halieti* (MZ329386, 1,774 bp)	98.74	*Milvus milvus*	Czech Republic
*S*. *neurona* SN5 (U07812, 1,803 bp)	98.68	*Equus caballus*	United States
*S*. *lutrae* (KM657769; 1,804 bp)	98.62	*Lutra lutra*	Norway
*S*. *moreliae* sp. nov (OQ296417; 1,472 bp)
*28S* rRNA
*Sarcocystis* sp. (MW349707; 1,509 bp)	95.41 (99)	*Aegolius funereus*	Finland
*S*. *wenzeli* (MT756986; 3,279 bp)	95.26	*Gallus gallus*	China
*S*. *neurona* (AF092927; 3,281 bp)	95.20	*E*. *caballus*	United States
*S*. *speeri* (KT207460; 1,522 bp)	95.19 (99)	*D. albiventris*	Argentina
*S*. *halieti* (MF946610; 1,504 bp)	95.16	*H. albicilla*	Norway
*S*. *lari* (JQ733509, 1,434 bp)	95.07 (96)	*Larus marinus*	Lithuania
*S*. *lutrae* (KM657771, 1,521 bp)	94.69	*L*. *lutra*	Norway

*Sarcocystis moreliae* sp. nov. (OQ295898; 1,037 bp)			
*Cox1*			
Species (sequence number; base pairs)	Similarity (query cover)^*^ %	Host species	Country
*Sarcocystis* sp. (MW074129; 987 bp)	98.78 (95)	*Hakaria simonyi*	Yemen
		*Trachylepis socotrana*	
*S*. *pantherophisi* (KU891603; 1,015 bp)	97.63 (97)	*Pantherophis alleghaniensis*	United States
*Sarcocystis* sp. (MW489293, 1,060 bp)	97.40	*A*. *funereus*	Finland
*S*. *falcatula* (MZ665257, 1,002 bp)	97.31 (96)	*Trichoglossus moluccanus*	United States
*S*. *strixi* (MF162317, 1,045 bp)	97.30	*Strix varia*	United States
*S*. *speeri* (KT207461; 1,057 bp)	97.30 (99)	*D. albiventris*	Argentina
*S*. *halieti* (MZ332969; 1,019 bp)	97.25 (98)	*Sturnus vulgaris*	Czech Republic
*S*. *lari* (MF596283, 1,053 bp)	97.20	*L. marinus*	Lithuania
*S*. *lutrae* (KM657808, 1,092 bp)	97.20	*L*. *lutra*	Norway
*S*. *moreliae* sp. nov (OQ296416; 1,016 bp)
ITS1 region
*S*. *halieti* (MZ333538, 992 bp)	99.01 (17)	*S*. *vulgaris*	Czech Republic
*Sarcocystis* sp. (MW373964; 1,294 bp)	92.86 (20)	*A*. *funereus*	Finland
*S*. *speeri* (KT207458, 1,190 bp)	91.18 (20)	*D*. *albiventris*	Argentina
*S*. *lutrae* (KM657805, 1,184 bp)	90.54 (23)	*L*. *lutra*	Norway
*S*. *cristata* (MT676453, 1,090 bp)	86.87 (25)	*Corythaeola cristata*	Central African Republic
*S*. *wenzeli* (MT756994, 1,186 bp)	86.36 (27)	*G*. *gallus*	China

^*^Query cover, 100% where not mentioned.

The sequence of the new species at *28S* rRNA contained single nucleotide polymorphisms (SNPs) at positions 52 (C/T) and 669 (A/G). This sequence was most similar to *Sarcocystis* sp. in the Tengmalm’s owl and *S*. *lari* in the great black-backed gull, *S*. *wenzeli* Wenzel, Erber, Boch et Schellner, 1982 in the chicken, *S*. *neurona* in the horse, *S*. *speeri* in *D. albiventris*, *S*. *halieti* in *H. albicilla*, and *S. lutrae* in *L. lutra* (see Table 1 for details).

The *cox1* sequence was most similar to *Sarcocystis* sp. in *Hakaria simonyi* and *Trachylepis socotrana*, *S*. *pantherophisi* (3) in the Eastern rat snake, *Sarcocystis* sp. in *A. funereus*, *S*. *falcatula* Stiles, 1893 in the rainbow lorikeet, *S*. *strixi* Verma, Rosypal von Dohlen, Mowery, Scott, Cerqueira-Cézar, Rosenthal, Dubey et Lindsay, 2017 in the barred owl, *S*. *speeri* in *D. albiventris*, *S*. *halieti* in the common starling, *S*. *lari* in the great black-backed gull, and *S. lutrae* (KM657808, 1,092 bp) in *L. lutra* (see Table 1 for details).

Amplification and sequencing of the ITS1 region were successful, and a representative sequence showed only one SNP at position 329, one double peak with A dominant over G, with no intraspecific variability for this marker. This sequence was similar to *S*. *wenzeli* in *G. gallus*, *S. cristata* Máca et González-Solís, 2021 in the great blue turaco, *S*. *halieti* in *S. vulgaris*, *S. lutrae* in *L. lutra*, *Sarcocystis* sp. in *A. funereus*, and *S*. *speeri* in *D. albiventris* (see Table 1 for details).

**Figure 4:**
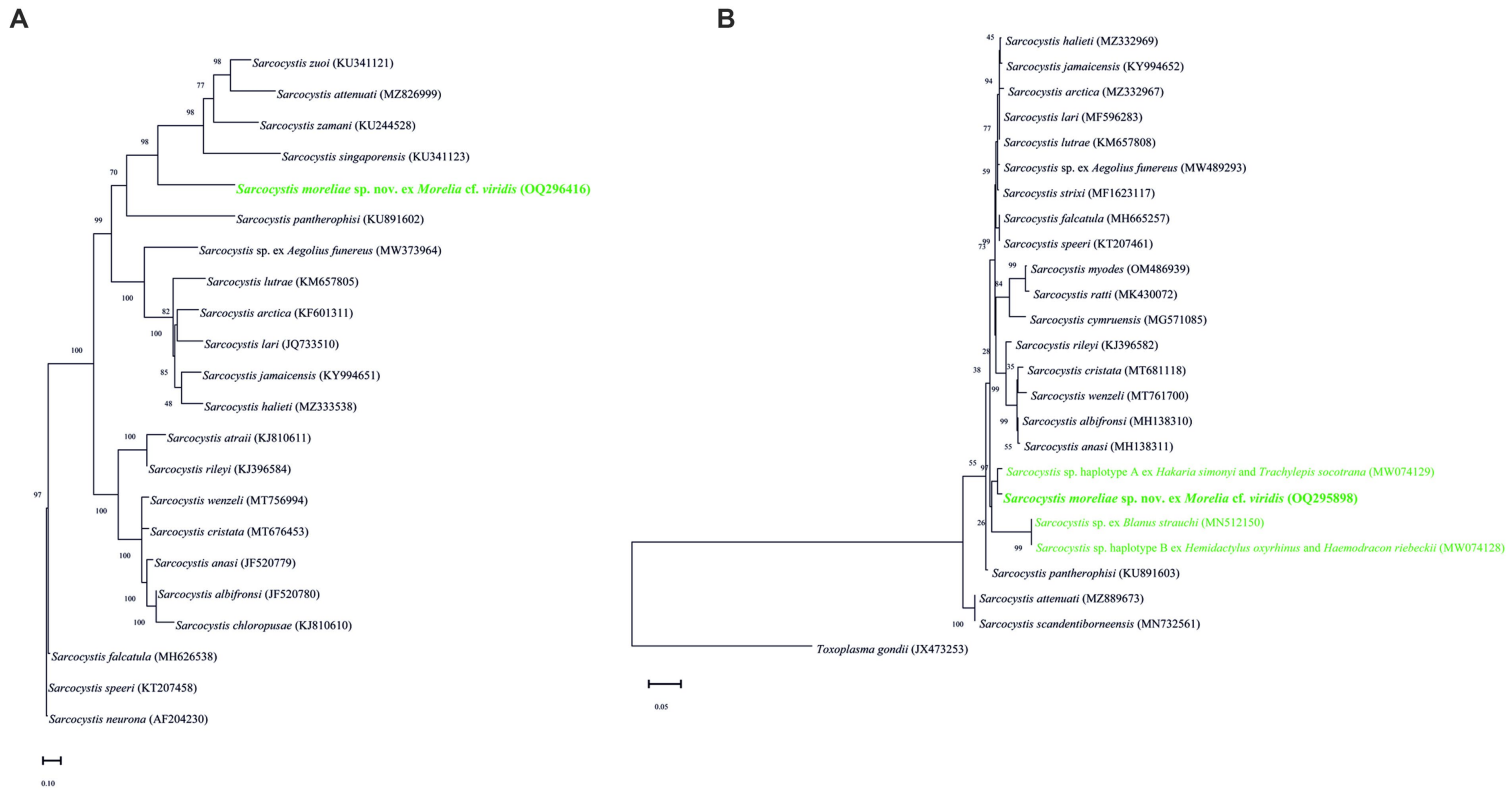
Phylogenetic tree of the species of *Sarcocystis*; those in green are for reptiles being used as intermediate hosts, based on **(A)** ITS1 and **(B)** *cox1* loci sequences. The numbers on phylogenetic trees represent bootstrap values based on 1,000 replications, while those after *Sarcocystis* species are GenBank accession numbers.

The topologies and phylogenetical relationships between *S*. *moreliae* sp. nov. with its congeners depended on the availability of sequences. The phylogenetic trees based on *18S* rRNA, *28S* rRNA, and *cox1* genes grouped the new species along with *Sarcocystis* spp. in *Ha*. *simonyi* and *T*. *socotrana*, as well as with other species using reptiles as IHs, such as *Sarcocystis* spp. in *Hemidactylus oxyrhinus* Boulenger and *Haemodracon riebeckii* (Peters) at *28S* and *cox1* genes (Figures 3A,B, 4B). In particular, and due to the higher availability of sequences in the *18S* rRNA gene, the tree displayed a larger branch where several unnamed *Sarcocystis* using reptiles as IHs and DHs were included (Figure 3A). On the other hand, the tree of the ITS1 region clearly isolated *S*. *moreliae* sp. nov. in a single clade within a group of species parasitizing snakes as DH (Figure 4A). None of the unnamed *Sarcocystis* used in the *18S* rRNA tree were so far sequenced at the ITS1 region, so its comparison was not possible.

## Discussion

Some *Sarcocystis* have so far used six snake species as IHs, excluding *S*. *moreliae* sp. nov. Of them, *S*. *atractaspidis* in *A. leucomelas* seems to belong to a different genus (e.g., *Besnoitia* Henry; see (16)), since its description was scarce and apparently incomplete and illustrations are missing (see (17)). On the other hand, the sarcocyst of *S*. *pythonis* in *M. spilota* and that of the new species in *M*. cf. *viridis* were similar in size (1,100 vs. 1,118 μm) and came from a congeneric snake species (see (18)), although *S*. *pythonis* is considered *species inquirenda* due to the lack of its molecular characterization. The sarcocysts of *Sarcocystis* sp. in *C*. *d*. *terrificus* also have septa, wall type 1, although slightly dissimilar in size, and with a thicker cyst wall than *S*. *moreliae* sp. nov. (2,000 μm × 50–150 μm; 1.00 vs. 0.55 μm, respectively; see (19)). Moreover, it was molecularly analyzed, but the sequence was not uploaded in GenBank. The sarcocysts found in *L. diadema*, *M. monspessulanus* (20), and *P. schokari* (21) were molecularly analyzed (*18S* rRNA), but their morphological descriptions are missing. Thus, the morphological comparison between the new species and those using snakes as IHs is practically impossible, coupled with the ineffectiveness of using the morphology of developmental stages to separate species. Therefore, counting on a detailed morphological description, complemented with a good molecular characterization (high-quality sequences with a large number of base pairs) and their availability will help with the phylogenetic analysis and clear identification of these protozoans.

The *18S* rRNA sequence of the new species matched completely with those of two unnamed *Sarcocystis* species in *L. diadema* and *V*. *s*. *macromaculatus* from Oman and Malaysia, respectively. However, it is difficult to say whether they belong to the same species since the latter two have not been phylogenetically analyzed (*18S* rRNA partially sequenced, no other loci), and morphological descriptions are lacking. In the case of *V*. *s*. *macromaculatus*, the site of infection, developmental stages (sarcocysts, sporocysts), and the role of the hosts as IHs or DHs are unknown. Moreover, the *18S* rRNA sequence length of *Sarcocystis* sp. in *L. diadema* was too short (588 bp; see (20)), and that in *V*. *s*. *macromaculatus* was only deposited in GenBank without formal publication. Interestingly, these two unnamed *Sarcocystis* have been recently grouped with *Sarcocystis* sp. using the California kingsnake (*L. californiae*) from Gran Canaria as a DH, although at a slightly lower percentage (99.7 and 99.3%, respectively) and with a short sequence (532 bp; see (22)). Thus, we consider our finding to be a new species and encourage the examination of the three unnamed *Sarcocystis* to elucidate their taxonomic status.

At *28S* rRNA and *cox1* loci, the new species was not 100% the same as any other *Sarcocystis* species, although in both trees, it seems to be a sister species of four unnamed *Sarcocystis* found in other reptiles of the families Blanidae (*Blanus strauchi* Bedriaga), Gekkonidae (*H. oxyrhinus*), Phyllodactylidae (*Hae*. *riebeckii*), and Scincidae (*Ha*. *simonyi*, *T*. *socotrana*). These unnamed species were closely related to other unnamed *Sarcocystis* that used lizards, lacertids, geckos, and reptiles as IHs (e.g., *M. monspessulanus*, *P. schokari*; see (23)), but they are all different from *S*. *moreliae* sp. nov. The role of various reptile species as IHs of *Sarcocystis* is still unknown but represents a possibility after considering their phylogenetic relationships. The use of the *cox1* gene to delineate species of *Sarcocystis* in snake hosts has already been proven, although the number of sequences at this locus is still small (24).

On the other hand, the similarity of the new species with its congeners at the ITS1 region was very low, but it formed a group with species using snakes as DHs, namely *S*. *attenuati* Hu, Sun, Guo, Zeng, Zhang et Tao, 2022, *S*. *pantherophisi*, *S. singaporensis* (Zaman et Colley, 1975), *S*. *zamani* Beaver et Maleckar, 1981, and *S*. *zuoi* Hu, Ma, et Li, 2005. Unfortunately, no *Sarcocystis* species using reptiles as IHs have been sequenced at this genetic region, so a comparison of the new species with others is impossible, and the use of this locus to separate species occurring in reptiles is still uncertain. The *18S* rRNA gene has been useful for the discrimination of sarcocystid protozoans (24), although the multigene phylogenetic analysis might be more robust in resolving relationships among species.

Since data on *Sarcocystis* using snake species of the genus *Morelia* as IHs are not available, of those in *M. viridis* as DHs that have been molecularly analyzed (i.e., KC201639 and KC201640), the new species clearly differs phylogenetically from them and forms a different clade. In fact, the above-mentioned sequences showed a close phylogenetic relationship with *S. singaporensis* and *S*. *zamani*, as well as being grouped along with other *Sarcocystis* species using snakes as DHs, as already stated by Moré et al. (5). These authors also mentioned that both sequences might either represent a known species of *Sarcocystis*, whose sequences are unavailable, or a new taxon. Whereas Wassermann et al. (25) pointed out that *S. singaporensis* is widely distributed in the Indo-Australian Archipelago and is probably frequently present in the green tree python in New Guinea, our results showed that this is not completely true, since other *Sarcocystis* spp. might also be present. Thus, *M. viridis* acts as IH and DH for at least three apparently different species of *Sarcocystis*, and all future parasitological examinations of this snake should include a review of feces and the intestinal tract, as well as the skeletal muscles in order to determine their possible presence.

None of the *Sarcocystis* species found in reptiles to date produce clinical illness. However, the present report of a new species of this genus in *M*. cf. *viridis* is important from diagnostic and epidemiological points of view because the parasite needs to find a definitive host to accomplish its life cycle. Unfortunately, only one green tree python was examined, and it is impossible to evaluate the impact of infection on other snakes acting as IHs, even for the one described here. This is an important finding, especially in connection with captive breeding, as this species is a great favorite of breeders worldwide, and with the importation of green tree pythons from areas where the complete life cycle can be realized, the risk of deterioration of the overall host condition is possible. This may be subject to further monitoring.

Snakes are considered potential carriers of human pathogens (see (25)). For example, *S*. *nesbitti* Mandour, 1969 uses snakes as DHs and could accidentally infect humans (see (26)) when the latter feed on food or water contaminated with sporocysts. However, when snakes act as IH, sporocysts might also be transmitted to humans when they eat improperly cooked sarcocyst-infected meat of the IH. Therefore, the presence of the new species should be monitored, especially in those places where the meat of snakes is used for human consumption.

The life cycle of *S*. *moreliae* sp. nov. is unknown, but its DHs might be monitors, birds, birds of prey (27, 28), quolls, or even other snakes. The black butcherbird (*Cracticus quoyi*) is distributed in Australia and New Guinea and is considered to be one of the most important predators of yellow individuals of *M. viridis* and might act as a DH of a *Sarcocystis* species. However, there are no records of protozoans in the feces of this bird species, except that of the informal report of *Isospora* sp. in *C. nigrogularis* from Queensland by O’Donoghue (29) at a conference.

As described above, there is only one case worldwide where a *Morelia* snake (*M. spilota*) was reported as an IH of a *Sarcocystis* species. Unfortunately, this was not molecularly analyzed, and its comparison with the new species is impossible. Therefore, this is the first morphological and genetic characterization of a *Sarcocystis* species in the *Morelia* genus and *M. viridis,* in particular, using four loci (*18S* rRNA, *28S* rRNA, ITS1, and *cox1*). This kind of genetic analysis is mandatory for distinguishing between members of this protozoan species since the morphologies of their developmental stages are rather subjective and have low taxonomic relevance.

## Data availability statement

The datasets presented in this study can be found in online repositories. The names of the repository/repositories and accession number(s) can be found below: https://www.ncbi.nlm.nih.gov/genbank/, OQ296418, OQ296417, OQ296416, and OQ295898.

## Author contributions

OM conceived and designed the study and performed analyses. OM and DG-S wrote the manuscript. All authors contributed to the article and approved the submitted version.

## Funding

Open access funding was provided by the Faculty of Agrobiology, Food and Natural Resources, Czech University of Life Sciences Prague.

## Conflict of interest

The authors declare that the research was conducted in the absence of any commercial or financial relationships that could be construed as a potential conflict of interest.

## Publisher’s note

All claims expressed in this article are solely those of the authors and do not necessarily represent those of their affiliated organizations, or those of the publisher, the editors and the reviewers. Any product that may be evaluated in this article, or claim that may be made by its manufacturer, is not guaranteed or endorsed by the publisher.

## Footnotes

^1^ http://www.ncbi.nlm.nih.gov/pubmed

^2^ http://mafft.cbcr.jp/aligment/server/index.html

## Abbreviations

bp: Base pairs
cox1: Cytochrome c oxidase subunit 1
DNA: Deoxyribonucleic acid
ITS1: Internal transcribed spacer 1
MAFFT: Multiple alignment using Fast Fourier Transform
rRNA: Ribosomal ribonucleic acid
